# Ethanol tolerance in engineered strains of *Clostridium thermocellum*

**DOI:** 10.1186/s13068-023-02379-z

**Published:** 2023-09-14

**Authors:** Daniel G. Olson, Marybeth I. Maloney, Anthony A. Lanahan, Nicholas D. Cervenka, Ying Xia, Angel Pech-Canul, Shuen Hon, Liang Tian, Samantha J. Ziegler, Yannick J. Bomble, Lee R. Lynd

**Affiliations:** 1 Thayer School of Engineering at Dartmouth College, Hanover, NH 03755 USA; 2 Center for Bioenergy Innovation, Oak Ridge National Laboratory, Oak Ridge, TN 37830 USA; 3 National Renewable Energy Laboratory, Golden, CO 80401 USA; 4 Present Address: College of Chemical and Biological Engineering, Zhejiang University, Hangzhou, China; 5 Present Address: Youth Olympic Village, #1-1-602, Jianye District, Nanjing, Jiangsu China

**Keywords:** Whole genome sequencing, Next-generation sequencing, Tolerance, Biofuel, *Hungateiclostridium thermocellum*, *Ruminiclostridium thermocellum*, *Acetivibrio thermocellus*, *AdhE*, *Alcohol dehydrogenase*, *Acetaldehyde dehydrogenase*

## Abstract

*Clostridium thermocellum* is a natively cellulolytic bacterium that is promising candidate for cellulosic biofuel production, and can produce ethanol at high yields (75–80% of theoretical) but the ethanol titers produced thus far are too low for commercial application. In several strains of *C. thermocellum* engineered for increased ethanol yield, ethanol titer seems to be limited by ethanol tolerance. Previous work to improve ethanol tolerance has focused on the WT organism. In this work, we focused on understanding ethanol tolerance in several engineered strains of *C. thermocellum*. We observed a tradeoff between ethanol tolerance and production. Adaptation for increased ethanol tolerance decreases ethanol production. Second, we observed a consistent genetic response to ethanol stress involving mutations at the AdhE locus. These mutations typically reduced NADH-linked ADH activity. About half of the ethanol tolerance phenotype could be attributed to the elimination of NADH-linked activity based on a targeted deletion of *adhE*. Finally, we observed that rich growth medium increases ethanol tolerance, but this effect is eliminated in an *adhE* deletion strain. Together, these suggest that ethanol inhibits growth and metabolism via a redox-imbalance mechanism. The improved understanding of mechanisms of ethanol tolerance described here lays a foundation for developing strains of *C. thermocellum* with improved ethanol production.

**Supplementary Information:**

The online version contains supplementary material available at 10.1186/s13068-023-02379-z.

## Introduction

Cellulose is a plentiful and renewable resource that can allow the production of biofuels with zero or even negative CO_2_ emissions. *Clostridium thermocellum* is a promising candidate for cellulosic biofuel production due to its native ability to consume cellulose [1]. Strains of *C. thermocellum* have been engineered to produce ethanol at titers of 25–30 g/L [2–4], however, this is too low for commercial application.

Previously, we have shown that ethanol tolerance appears to limit ethanol titer, based on the observation that the sum of added and produced ethanol has a constant value of about 22 g/L, which is very close to the maximum ethanol titer of that strain [2]. Several groups (including us) have shown that WT *C. thermocellum* can be adapted to grow in the presence of > 50 g/L ethanol [5–7]. However, no work has been done adapting strains of *C. thermocellum* engineered for increased ethanol production to also tolerate more ethanol.

We initially considered strains from two different engineering lineages. One lineage focused on deleting competing pathways for carbon and electron flux (i.e., acetate, lactate, hydrogen, and formate) [2]. This strain (LL1210) was able to produce ethanol from cellulose at a titer of 22.4 g/L but grew poorly. Another lineage focused on heterologous expression in *C. thermocellum* of the ethanol production pathway from the proficient ethanol producing organism, *Thermoanaerobacterium saccharolyticum* [8]. This pathway included pyruvate ferredoxin oxidoreductase (*pforA*), NADH-dependent reduced ferredoxin:NADP^+^ oxidoreductase (*nfnAB*), ferredoxin (*fd*), a mutant bifunctional aldehyde dehydrogenase (*adhE*^*G544D*^), and a monofunctional NADPH-linked alcohol dehydrogenase (*adhA*) [3]. The *C. thermocellum* strain expressing this pathway (LL1570) was able to produce ethanol from cellulose at a titer of 25 g/L and grew well.

In the LL1570 lineage, we made some additional modifications we hoped would further increase ethanol titer and/or tolerance. Previously, we have observed that strains engineered for increased ethanol tolerance [9] or decreased acetate production accumulate lactate [10]. We, therefore, deleted the *ldh* gene to prevent lactate accumulation [11], resulting in strain LL1592. Previously we also observed that ethanol production inhibited glycolysis at the glyceraldehyde-3-phosphate dehydrogenase (GAPDH) reaction, and that the GapDH enzyme from *T. saccharolyticum* was less sensitive to inhibition by NADH, compared to the native *C. thermocellum* enzyme [12]. Heterologous expression of the *gapDH* gene from *T. saccharolyticum* in *C. thermocellum* resulted in strain LL1707. As an alternative to *gapDH*, we expressed the *gapN* gene from *Sulfolobus solfataricus*, since we had previously performed thermodynamic analysis that suggested that the non-phosphorylating GapN enzyme might allow increased ethanol titer in *C. thermocellum* [13].

**Fig. 1:**
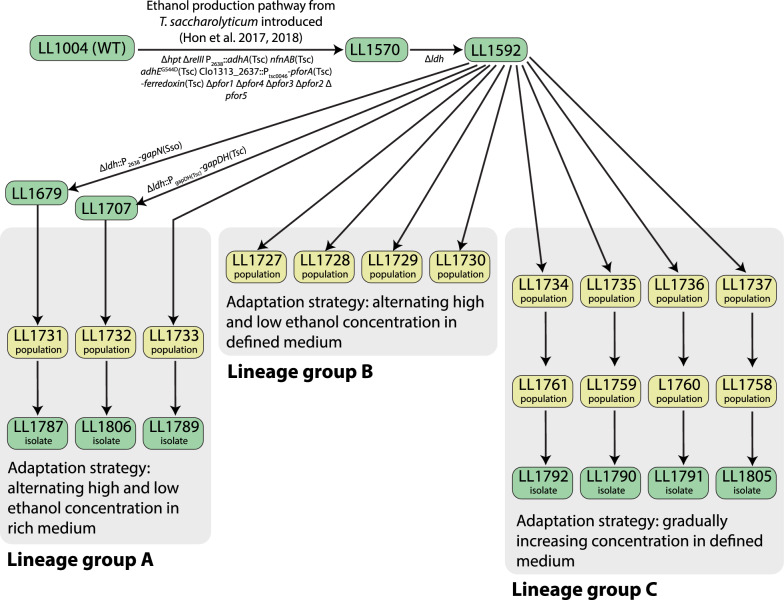
Strain lineage diagram. Green boxes represent clonal isolates, yellow boxes represent adapted populations

To understand the effect of growth medium on ethanol tolerance, we performed experiments in either chemically defined (MTC-5 [14]) or rich (CTFUD [15]) medium. For the strains expressing alternative glycolytic enzymes, we performed adaptation in a rich medium to give the cells more metabolic flexibility to use the heterologous genes. For the other strains, we performed adaptation in a defined medium to restrict metabolic flexibility and prevent the accumulation of auxotrophic mutations (Fig. 1).

To understand the effect of ethanol adaptation strategy on ethanol tolerance, we performed adaptation with two different strategies: alternating high and low concentrations, or continuous increase (Table 1). For the alternating strategy, we grew cells on alternating high and low concentrations of ethanol. This strategy has been proposed as a way to select for constitutive expression of a desired trait, and has been used successfully in prior *C. thermocellum* ethanol adaptation experiments [7]. In cases where that did not work, we switched to the continuously increasing approach.

**Table 1:** Selection strategies

Lineage group	Genotype	Medium	Ethanol addition strategy
A	*T. saccharolyticum* ethanol production pathway, and altered glycolytic genes	Rich	Alternating high and low
B	*T. saccharolyticum* ethanol production pathway	Defined	Alternating high and low
C			Continuously increasing

We then set out to adapt these engineered strains of *C. thermocellum* for increased ethanol tolerance. We hypothesized that if tolerance was limiting titer, increasing tolerance would allow further increases in titer. We were also interested to better understand how the genetic modifications in our engineered strains would affect adaptation to ethanol.

## Results

### Initial ethanol titer tests

**Fig. 2:**
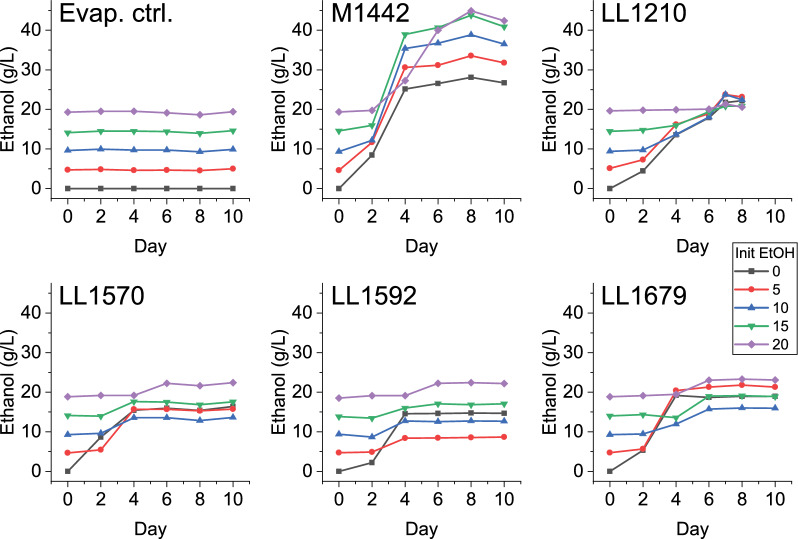
Ethanol titer in the presence of different concentrations of added ethanol. Four engineered strains of *C. thermocellum* (LL1210, LL1570, LL1592, and LL1679) were compared to an uninoculated control (Evap. ctrl.), and an ethanol-tolerant strain of *Thermoanaerobacterium saccharolyticum* (M1442). The data for strain LL1210 are from [2] and is included for purposes of comparison. Cells were grown in MTC defined medium with 50 g/L cellobiose in sealed glass bottles. WT *C. thermocellum* was not included since it is not able to consume 50 g/L cellobiose in the absence of pH control. Total ethanol titer is shown, representing the sum of initially present and produced ethanol. Strain genotypes are described in more detail in Fig. 1 and Table 2

Previously, we had shown that ethanol tolerance was limiting ethanol titer in strain LL1210 [2]. To understand whether tolerance also limited ethanol titer in our other engineered strains, we performed the same experiment with these strains. In all the engineered strains of *C. thermocellum*, ethanol production was strongly inhibited by added ethanol, with production approaching zero at the highest added ethanol concentration of 20 g/L (Fig. 2). This contrasts sharply with our ethanol producing strain of *T. saccharolyticum* (M1442), which readily converted all 50 g/L of the cellobiose substrate initially present into 27 g/L ethanol, and this was only slightly reduced (to 25 g/L) in the presence of 20 g/L initial ethanol.

### Adaptation

**Fig. 3:**
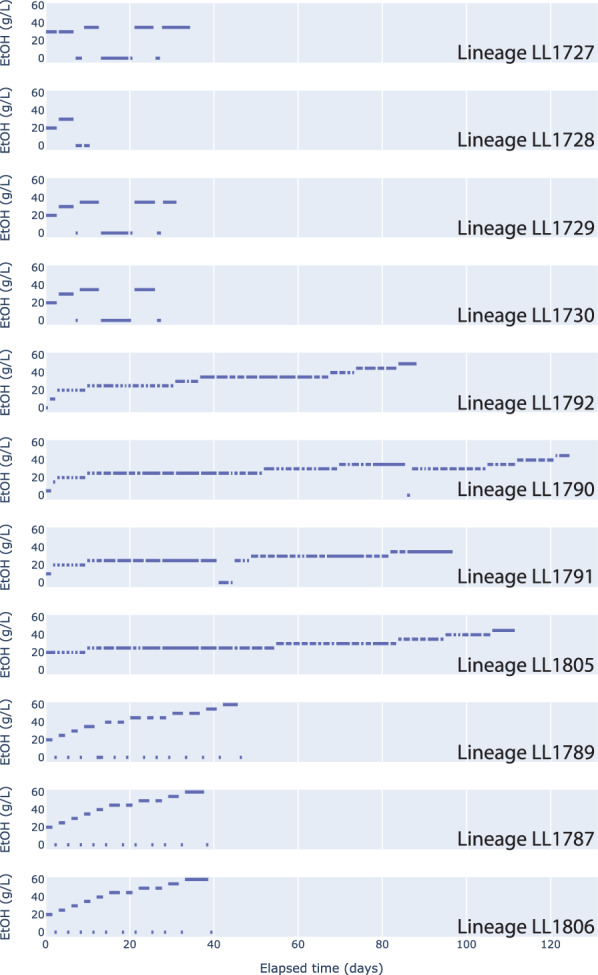
History of serial transfers for each of the lineages described in this work. Blue bars represent the duration of each serial transfer

After demonstrating that ethanol production was limited by ethanol titer in all the engineered strains of *C. thermocellum* we tested (strains LL1570, LL1592, and LL1679), we proceeded with ethanol adaptation experiments. (Note: we did not perform adaptation on strain LL1210 since it still grew slowly despite previous adaptation [2]). These un-adapted strains were able to grow in the presence of 20 g/L ethanol in all conditions, and we therefore used this as a starting ethanol concentration for almost all of our adaptation work (the LL1790, LL1791, and LL1792 lineages were started at lower ethanol concentrations, but the ethanol concentration was increased to 20 g/L after just a few transfers) (Fig. 3).

Since, we had previously used the alternating high–low adaptation strategy to increase ethanol tolerance in *C. thermocellum* [7], we started with that approach for both the rich and defined medium conditions. Strains grown in rich medium were able to grow in the presence of 60 g/L ethanol after a relatively brief period (40 days) of adaptation.

In defined medium, the alternating strategy for ethanol adaptation caused very long lag phases and was abandoned. Using the continuously increasing strategy for ethanol adaptation in defined medium, the maximum ethanol tolerance achieved was 40 g/L. The experiment was stopped due to logistical limitations of the COVID 19 pandemic, so it is not known whether additional transfers would have further increased ethanol tolerance.

### Genome resequencing

**Fig. 4:**
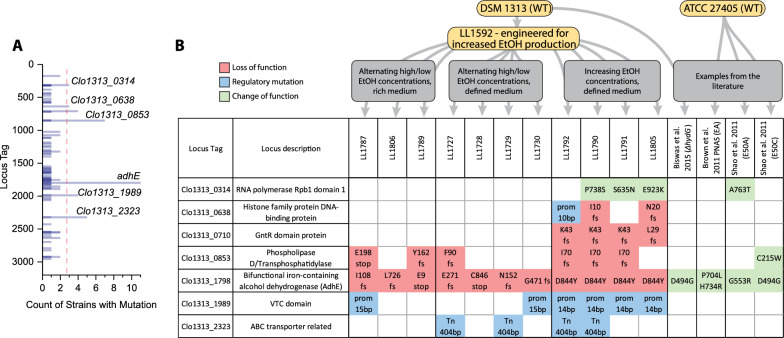
Analysis of mutations. Panel A shows the count (frequency) of strains with mutations at each locus. Eleven strains were analyzed for this analysis, consisting of the final isolates or populations for each of the three lineages. These strains include LL1787, LL1806, LL1789, LL1727, LL1728, LL1729, LL1730, LL1792, LL1790, LL1791, LL1805. Mutations occurring in three or more strains are described in panel B. Mutations affecting a coding sequence are described by the one-letter amino acid abbreviation for the native residue, the amino acid position, and the mutated residue. Abbreviations include stop: stop codon, fs: frameshift, prom: mutation in promoter region, Tn: transposon insertion. Mutations are color-coded to indicate expected function (see key in figure). Additional mutations from other published work describing ethanol adaptation of *C. thermocellum* is included for context [6, 7, 16]

To identify genetic modifications that had occurred during adaptation, we performed whole-genome sequencing on the adapted strains. A complete table of mutations is included in the supplement (Additional file 1: Table S1). To identify signatures of convergent evolution, we looked for genes that had accumulated mutations across several different strains (Fig. 4).

#### AdhE (Clo1313_1798)

The most frequently mutated gene was *adhE*. All 11 of the lineages sequenced had mutations at this locus. Most of the mutations in the *adhE* gene were frameshift (fs) or premature stop codons (stop) that would be expected to eliminate activity. One mutation, D844Y, appeared in several parallel lineages from the same adaptation strategy (strategy C).

**Fig. 5:**
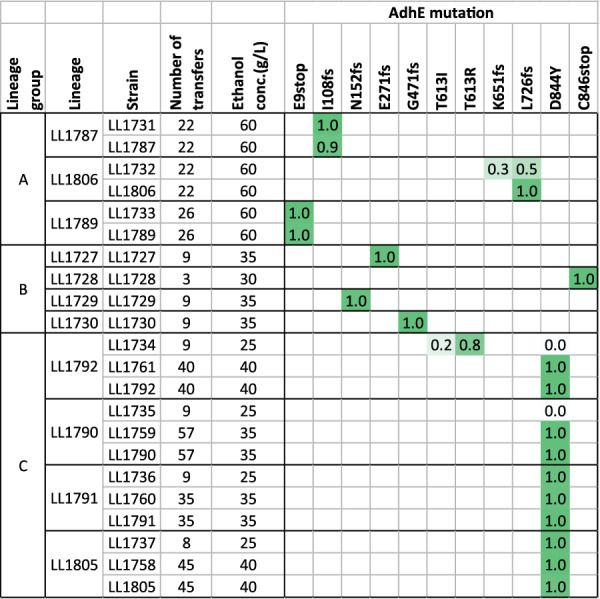
Appearance of AdhE mutations in different strain lineages. This figure shows when each AdhE mutation appeared in its respective lineage. No AdhE mutations were present in the parent strain. The ethanol concentration present during the growth of each strain is noted. For each mutation, the fraction of reads supporting the mutation is indicated

The multiple occurrences of the D844Y mutation could be explained either by its presence in the parent strain (LL1592) or by convergent evolution. The D844Y mutation is caused by a C → A mutation at position 2,096,168 of the genome. In the parent strain, 0 of 76 reads have an A nucleotide, suggesting that if this mutation was present in the parent strain culture, it was present at a frequency of < 1%. Furthermore, we can see that in the LL1792 and LL1790 lineages, the D844Y mutation was not present in either population after 9 transfers (Fig. 5), providing additional evidence that this mutation appeared independently in each of the lineages.

It is also noteworthy that the native *adhE* was the only ADH gene that was targeted for mutation. Strain LL1592 lineage carries two ADH genes from *T. saccharolyticum* (*adhE*^G544D^ and *adhA*), mutations were not observed in these genes in any of the adapted strains. *C. thermocellum* has five other genes annotated as alcohol dehydrogenases (Clo1313_0076, Clo1313_0166, Clo1313_1827, Clo1313_1833, and Clo1313_2130). In this set of genes, there was only a single mutation, A151V in Clo1313_1827. This mutation only appeared in a single lineage (LL1732 population and LL1806 isolate).

#### Phospholipase D (also known as cardiolipin synthase, Clo1313_0853)

The Clo1313_0853 gene appears to be a target of convergent evolution for strains of *C. thermocellum* adapted to grow in the presence of added ethanol, although the signature is weaker than for *adhE*. In this gene, five different alleles were identified across seven lineages (out of 11 total lineages studied), including at least one from each of the lineage groups A, B, and C (Fig. 1). In all five cases, the mutation was a loss-of-function mutation (frameshift or stop codon). Mutations in this gene have also been found in other strains of *C. thermocellum* adapted to ethanol [7] and n-butanol [17]. Despite several attempts, we were unable to create a targeted disruption of the Clo1313_0853 locus in the LL1592 parent strain.

#### Other genes

Signatures for convergent evolution were also found in RNA polymerase *rpoC* (Clo1313_0314), histone-family DNA binding protein (Clo1313_0638), a GntR transcriptional regulator protein (Clo1313_0710), a VTC domain protein (Clo1313_1989), and an ABC-transporter related protein (Clo1313_2323), however, these signals are generally weaker (fewer strains with the mutation, fewer lineage groups with the mutation, no examples in other *C. thermocellum* ethanol adaptation literature) than what was observed for *adhE* or Clo1313_0853.

### Effects on fermentation products

To understand the effect of adaptation for increased ethanol tolerance on ethanol production, we performed batch fermentations in the presence and absence of 10 g/L ethanol. We only performed fermentation experiments on strains from lineage groups A and C. We use high concentrations of substrate (50 g/L (146 mM) cellobiose), to maintain consistency with our adaptation conditions, and to allow observation of ethanol production in the presence of added ethanol. A complete table of fermentation data is presented in the supplement (Additional file 1: Table S2).

**Fig. 6:**
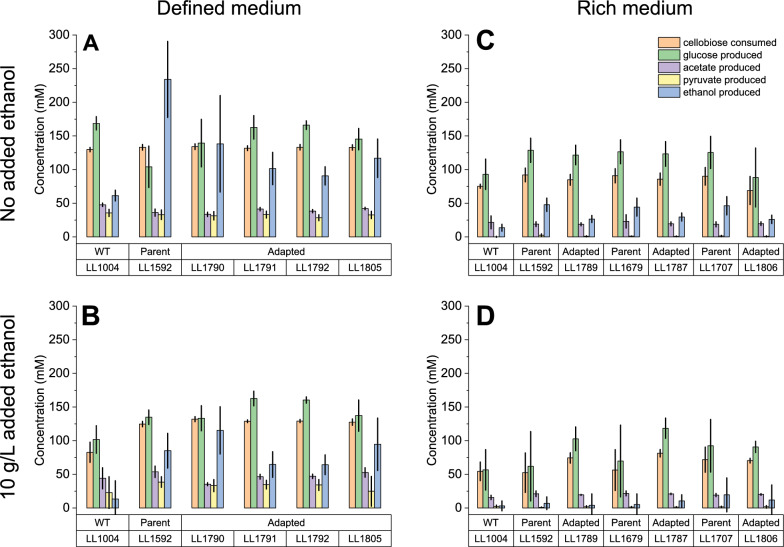
Fermentation profile of ethanol adapted strains in the presence and absence of added ethanol. For all fermentations, the cellobiose concentration was 50 g/L (146 mM). Panels **A** and **B** show cells grown in chemically defined medium (MTC-5). Panels **C** and **D** show cells grown in rich medium (CTFUD). The bottom two panels (**B** and **D**) show cells grown in the presence of 10 g/L added ethanol. In panels **C** and **D**, each adapted strain is next to its parent strain. Error bars represent one standard deviation, n = 3 biological replicates

The primary fermentation products were glucose, ethanol, acetate, and pyruvate (Fig. 6). In most cases, the majority of cellobiose (50–80%) was converted to glucose. This is commonly observed in *C. thermocellum* batch fermentations with high concentrations of substrate. It is not known whether this conversion takes place intracellularly or extracellularly. Carbon recovery was 90–98% on defined medium (MTC-5), and 78–94% on rich medium (CTFUD).

We observed three general trends with respect to ethanol production. (1) Increased ethanol tolerance did not result in increased ethanol production, and in some cases, even decreased production. (2) Addition of ethanol reduced ethanol production. (3) Strains grown in rich medium (CTFUD) produced less ethanol compared to strains grown in defined medium (MTC-5).

### Effect of adaptation on enzyme activity

**Fig. 7:**
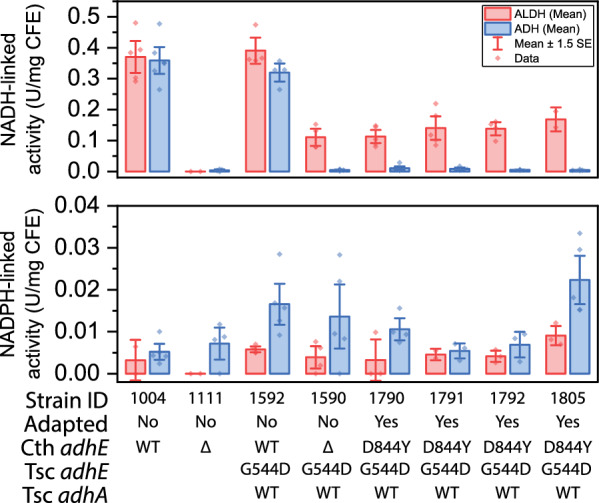
ALDH and ADH activity was measured in cell extracts with both NADH and NADPH as cofactors. Enzyme assays were performed in a 60 µl volume in a 384 well plate. Error bars represent one standard deviation, n ≥ 3. Strain LL1592 was constructed with a *T. saccharolyticum* AdhE containing a G544D mutation, which all descendant strains inherited

To study the effect of adaptation on enzyme activity, we focused on the strains from lineage group C (LL1790, LL1791, LL1792, and LL1805), all of which had the D844Y mutation. Since AdhE was the most common target of mutations, we measured ALDH and ADH activity. Since we have previously observed mutations in AdhE that affect its cofactor specificity [18], we measured both activities with both NADH and NADPH cofactors. In WT *C. thermocellum*, ADH activity is > 99% NADH-linked. In the LL1592 parent strain, expression of the *T. saccharolyticum adhA* gene results in NADPH-linked ADH activity (although levels are relatively low). The primary effect of adaptation appears to be a loss of NADH-linked ADH activity. NADH-linked ALDH activity also decreased (Fig. 7).

### Characterizing the D844Y mutation

**Fig. 8:**
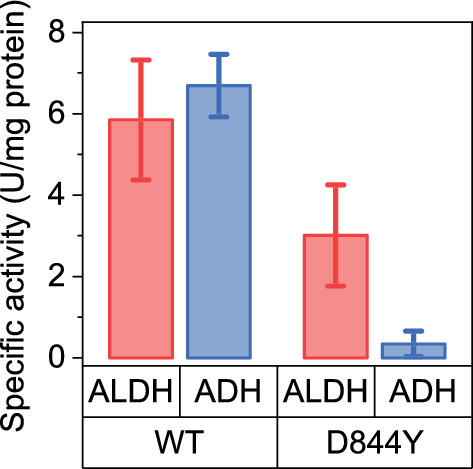
Characterization of a purified AdhE protein. The *C. thermocellum adhE* gene, with or without the D844Y mutation, was cloned, expressed, and purified in *E. coli* using a his-tag. The resulting protein was assayed for ALDH and ADH activity with NADH as the cofactor. Error bars represent one standard deviation n ≥ 3

To understand the effect of the D844Y mutation, the *adhE* gene from *C. thermocellum* carrying the D844Y mutation was cloned and expressed in *E. coli.* Activity was measured for both the ALDH and ADH reactions with both NADH and NADPH cofactors. No activity was detected with the NADPH cofactor for either the ALDH or ADH reaction. The mutation significantly reduced ADH activity, and slightly reduced ALDH activity (Fig. 8). Since AdhE is a bifunctional enzyme, the apparent decrease in ALDH activity may actually represent a measurement artifact. For the WT enzyme, each molecule of acetyl-CoA that is consumed can result in the consumption of either one or two molecules of NADH, depending on whether or not the acetaldehyde is further converted to ethanol, and the exact number is not known. If the ADH reaction is blocked, however, the reaction stoichiometry is fixed at one NADH per acetyl-CoA.

### Effects of adaptation on ethanol tolerance

**Fig. 9:**
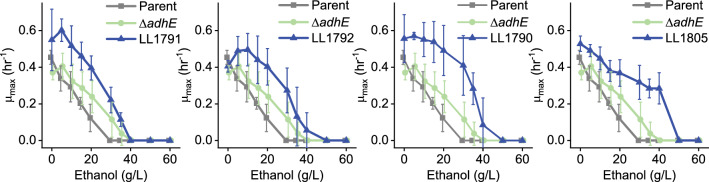
Adaptation of strains to added ethanol. Growth rate was measured in a 96-well plate with various concentrations of added ethanol in MTC-5 medium with 5 g/L cellobiose. For strains LL1790, LL1791, LL1792, and LL1805, the parent strain is LL1592. In strain LL1592, a targeted deletion of the *C. thermocellum adhE* resulted in strain LL1590. Error bars represent one standard deviation with 2–5 biological replicates. Traces are slightly offset along the x-axis to more clearly show error bars

To confirm that our adaptation increased ethanol tolerance, we measured the growth of strains in the presence of different concentrations of added ethanol (Fig. 9). Adaptation increased both the growth rate for a given ethanol concentration, and the maximum ethanol concentration at which growth could be initiated. All the adapted strains showed an increase in ethanol tolerance from the 20 g/L of the parent strain (LL1592) to 35–40 g/L for the adapted strains. This closely matches the ethanol tolerance observed during the adaptation work (Fig. 3).

To confirm the genetic basis for this increased ethanol tolerance we focused on understanding the effect of *adhE* mutations. Initially, we tried to reintroduce the D844Y mutation using recently-developed CRISPR-based tools [19]. Despite several attempts, we did not succeed. Since many of the *adhE* mutations were expected to completely inactivate the enzyme, we instead performed a targeted deletion of the *C. thermocellum adhE* in the parent strain (LL1592). Deletion of *adhE* could explain about half of the observed ethanol adaptation phenotype.

### Effects of rich medium on ethanol tolerance

**Fig. 10:**
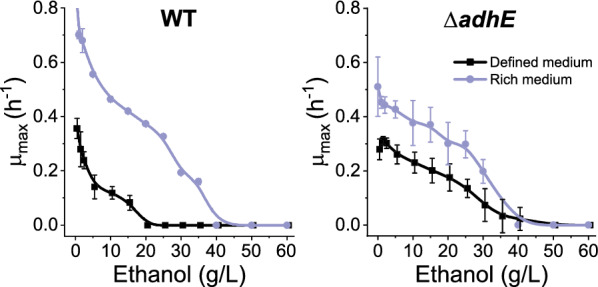
The interaction of rich medium and the *adhE* deletion on ethanol tolerance. Ethanol tolerance was measured for both WT and *adhE* deletion (strain LL1111) cells in either chemically defined (MTC-5) or rich (CTFUD) medium. Ethanol tolerance was determined from growth rate measurements in a 96-well plate format. Error bars represent one standard deviation from 2–5 biological replicates

In our initial adaptation experiments, we observed that strains grown in rich medium exhibited increased ethanol tolerance. Our genome resequencing work suggested that inactivation of *adhE* also increased ethanol tolerance. To study the interaction between the two effects, we measured ethanol tolerance of both the WT and *adhE* deletion strains in both rich and defined medium. Ethanol tolerance of the WT strain was substantially improved by growth in rich medium. By contrast, in the *adhE* deletion strain, rich medium had very little effect on ethanol tolerance (Fig. 10).

## Discussion

### The role of *adhE* in ethanol tolerance

Consistent genetic responses to ethanol stress have been difficult to find in many organisms. The genetic basis for ethanol tolerance in both *S. cerevisiae* and *E. coli* has been a topic of several studies over the past few decades [20–25]. It is commonly assumed that ethanol tolerance is a multi-gene trait, possibly involving hundreds of genes [20, 21, 26, 27]. Several groups have used systems biology tools to study the genetic basis for ethanol tolerance in *E. coli*. [23, 24, 27–31]. Genes associated with ethanol tolerance include those that participate in fatty acid biosynthesis [28, 32], peptidoglycan synthesis [23, 32], osmotic stress response [23, 28, 31, 32], the stringent response [29], heat shock [33], DNA repair [28], transcriptional machinery [30], aerobic respiration [23], and ethanol consumption [23, 31]. There is, however, remarkably little overlap in the specific genes identified in different studies, and individual genes typically exhibit small effects.

By contrast, the genetic response to ethanol stress in *C. thermocellum* is much more uniform. Mutations to the *adhE* gene are almost universally observed in strains adapted for increased ethanol tolerance. All 11 of the lineages we studied exhibited mutations in *adhE*, and these mutations occurred early in the adaptation process, often in the first few generations (Additional file 1: Table S1). Mutations at this locus have also been observed for both previous ethanol adaptation experiments with WT *C. thermocellum* [6, 7]. This locus was targeted regardless of variations in experimental details including: the presence [5] or absence [7] of chemical mutagenesis (including this work), in rich or chemically defined medium, and between different ethanol adaptation strategies (alternating high–low vs. constantly increasing). Furthermore, this effect has been observed by different experimenters in different laboratories. Thus, any explanation of ethanol tolerance in *C. thermocellum* must consider the role of *adhE*.

### The effect of AdhE mutations

Most of the observed AdhE mutations are loss-of-function mutations (frameshift or early stop codon), which would be expected to disrupt both ALDH and ADH activity. Several strains exhibited a D844Y mutation. The D844 residue is highly conserved—in a sequence alignment of 1138 *adhe* genes, aspartate is conserved across 99.6% of the sequences. Further, its proximity to the catalytic Fe atom in the ADH domain, suggests that it may play a role in catalysis. When analyzing the analogous residue in the *E. coli* AdhE cryo-EM structure (PDB ID 7BVP [34]), D839 is 11 Å from the catalytic Fe atom and 12 Å from the NAD^+^, in a clear binding pocket. Utilizing the mutagenesis tool to create a D-to-Y mutant introduces a bulkier side chain into this pocket, which explains the decrease in activity we have identified. Indeed, strains with the D844Y mutation have lost almost all the NADH-linked ADH activity present in the parent strain (Fig. 7). This was further confirmed by assaying purified AdhE^D844Y^ enzyme (Fig. 8). Mutations in AdhE can explain both the decrease in NADH-linked ADH activity and the decrease in ethanol production. Loss-of-function mutations in AdhE can explain about half of the ethanol tolerance phenotype. (Fig. 9).

Added ethanol does not appear to provide selective pressure for eliminating ALDH activity. All the mutations observed in *adhE* eliminated or reduced ADH activity. Some of them also eliminated ALDH activity, but no large difference in ethanol tolerance was observed. Thus, the primary effect of AdhE mutations appears to be the elimination of NADH-linked ADH activity. This explanation is consistent with all 11 of the lineages tested here, and at least one of the previously published examples (i.e. the AdhE^P704L H734R^ mutant) [6, 18].

One notable exception to this general pattern is the AdhE^D494G^ mutation observed by Shao et al. [7]. This mutation increases NADPH-linked ADH activity, but has no effect on NADH-linked ADH activity [18]. Interestingly, other *adh* genes in *C. thermocellum* were not targeted for mutations.

### The role of other mutations in ethanol tolerance

Five independent mutations were observed in the phospholipase D gene (Clo1313_0853), all loss-of-function mutations (frameshift of premature stop codon). These mutations were present in 7 of 11 adapted lineages. A mutation in this gene (corresponding to Cthe_1396 in C. thermocellum strain ATCC 27405) was also observed in one previous ethanol adaptation report [7].

Phospholipase D (also known as cardiolipin synthase) catalyzes the conversion of glycerophopholipids to phosphatidic acid, an important step in membrane biosynthesis. Changes in membrane composition in response to ethanol stress have been shown in *C. thermocellum* [40, 42] and other microbes [35, 36). However, it is not clear why disruption of this gene would lead to increased ethanol tolerance. Disruptions of cardiolipin synthase in *E. coli* do not have a detectable phenotype [37, 38].

### The tradeoff between ethanol tolerance and ethanol production

We observed a clear tradeoff between ethanol tolerance and ethanol production. Previously it was shown that strains adapted to tolerate high levels of ethanol produced higher yields of lactate and lower yields of ethanol [9]. This has been observed in other obligate anaerobes as well [39]. In this work, we also observed that ethanol production decreased. There are two components to this. An adaptive response related to the reduction in ADH activity due to mutations, and an inhibitory effect that further reduces ethanol production (Fig. 6). However, since all of the strains in this study are *ldh* deletion mutants, we did not observe an increase in lactate production. The other major pathway that *C. thermocellum* can use to eliminate excess NADH equivalents is H_2_ production. We did not measure H_2_ production directly, but production of H_2_ is typically associated with acetate production due to redox balance constraints. On defined medium, ethanol adapted strains showed an increase in acetate production relative to the parent strain (LL1592), but acetate was still produced at levels lower than that of the WT strain. Furthermore, the increased acetate production was not sufficient to account for the decrease in ethanol production. Thus, there must be some other reduced product that is formed by the ethanol adapted strains, however that product is not known. Understanding the underlying mechanism of this tradeoff is discussed in detail below.

### The importance of LDH activity for maintaining redox balance

In previous work, WT *C. thermocellum* has been adapted to tolerate 50 g/L ethanol [5, 7]. Strains with *ldh* deletions (all of the strains investigated in this work) could not be adapted to tolerate > 40 g/L ethanol. One confounding factor was the interruption of this work by the COVID 19 pandemic. It is possible that given additional time, further increases in ethanol tolerance would have been observed, however it seems that *ldh* deletion strains are generally more sensitive to ethanol than strains with functional *ldh* genes (compare the WT strain vs. LL1592, for example). The LL1111 *adhE* deletion strain has an S161R mutation which deregulates the *ldh* gene [11]. This may improve ethanol tolerance by providing alternative pathways for reducing excess NADH generated in glycolysis.

### The role of rich medium in ethanol tolerance

Rich medium allows higher ethanol tolerance compared to defined medium (Fig. 10). In previous work, adaptation of *C. thermocellum* to tolerate ethanol concentrations of 80 g/L has been reported on rich medium [40], whereas adaptation on defined medium resulted in strains that could tolerate only 50 g/L [7]. Rich medium appears to play a role in redox balance since it provides a beneficial effect for the WT strain, but not the *adhE* deletion strain.

### The mechanism of ethanol inhibition in *C. thermocellum*

Mechanisms of ethanol inhibition can be grouped into two main categories: chaotropic effects—stemming from the ability of ethanol to disrupt hydrogen bond networks (e.g. fluidization cell membranes, denaturation of protein and DNA, disruption of molecule binding, etc.), and metabolic effects—stemming from the participation of ethanol in the network of metabolic reactions. The ADH reaction connects ethanol to other reactions in metabolism and therefore plays a key role in metabolic mechanisms of ethanol inhibition. Since the ADH reaction involves redox cofactors, it mediates the ability of ethanol to perturb redox balance.

**Chaotropic mechanism** it has long been known that *C. thermocellum* is inhibited by ethanol at concentrations as low as 5 g/L [41]. Initial studies focused on the effect of changes in membrane lipid composition [40, 42], however long-term and short-term adaptation studies have shown conflicting results regarding the effect of ethanol on fatty acid chain length. A recent study looking at the interaction between growth temperature and ethanol inhibition found that ethanol tolerance was increased at lower growth temperatures [43]. Since ethanol and temperature both have chaotropic effects, this could indicate a role for chaotropicity in ethanol inhibition. However, the effect was relatively small, and disappeared in a strain where *adhE* was deleted.

**A redox imbalance mechanism** of ethanol inhibition has been described in detail for the thermophilic anaerobe *Thermoanaerobacter pseudethanolicus* 39E [39] (formerly *Clostridium thermohydrosulfuricum* [44]). In this mechanism, ethanol is consumed by the NADH-linked ADH reaction, increasing the NADH/NAD^+^ ratio. This increased NADH/NAD^+^ ratio blocks glycolysis at the GAPDH reaction. Mutations that eliminate NADH-linked ADH activity break the link between ethanol titer and the NADH/NAD^+^ ratio, increasing ethanol tolerance. Although Lovitt et al. did not determine the molecular mechanism of the loss of NADH-linked ADH activity, the concurrent loss of both ALDH and ADH activity in their ethanol-adapted strain strongly implicates mutations at the *adhE* locus (Teth_0206), since that is the only gene annotated to have ALDH activity.

This mechanism appears to be the primary cause of ethanol inhibition in *C. thermocellum* as well, based on several independent lines of evidence:

- As early as 1985, it was observed that addition of ethanol to *C. thermocellum* cultures causes an increase in hexose phosphate concentrations [45]. This finding was confirmed in several subsequent studies [12, 46], and the site of metabolic inhibition was narrowed down to a region surrounding the GAPDH enzyme [12].
- The elimination of NADH-linked ADH activity increases ethanol tolerance. This can be achieved by targeted deletion of the *adhE* gene (Fig. 9), or by point mutations that disrupt its function (Fig. 7).
- Deletion of the *ldh* gene makes it more difficult for strains to adapt to ethanol stress. In the absence of ethanol production, lactate production is one of the main mechanisms for balancing the NADH generated in glycolysis [11].
- The ability of rich growth media to improve ethanol tolerance in the WT strain, but not in the *adhE* deletion strain. Since *adhE* is not known to affect either membrane composition or osmotic stress, we would expect ethanol sensitivity due to either of these mechanisms to affect both WT and *adhE* deletion strains equivalently. Instead, however, we see that rich medium improves ethanol tolerance in the WT strain and has almost no effect on ethanol tolerance in the *adhE* deletion strain (Fig. 10). Since *adhE* is known to play a role in redox balancing [47], this suggests that the protective effect of rich medium may be due to its impact on redox.

The implications of this are that NADH-linked ADH activity is not compatible with NAD-linked GAPDH activity for high titer ethanol production. In this work, we attempted to test this hypothesis by expressing the non-phosphorylating GapN enzyme, however this did not result in any increase in ethanol tolerance compared to the parent strain. It is possible that the GapN enzyme is not functional in *C. thermocellum*. It is also possible that our use of rich medium diminished the redox imbalance associated with added ethanol, and that this may have masked any potential increases in ethanol tolerance from the strain expressing GapN.

### Remaining questions

The mechanism of ethanol inhibition in the *adhE* deletion strain is not fully known. Even though NADH-linked ADH activity has largely been eliminated in this strain, it is still inhibited by ethanol at concentrations > 40 g/L. This may be due to residual low levels of NADH-linked ADH activity or may be due to a non-metabolic mechanism of ethanol inhibition.

The mechanism of ethanol tolerance in the AdhE ^D494G^ mutant is not known. This mutant is the only example of an *adhE* mutation observed in a strain of *C. thermocellum* selected for increased ethanol tolerance [7] that does not reduce NADH-linked ADH activity. In this mutant NADH-linked ADH activity is unchanged, but NADPH-linked ADH activity is increased [18].

## Materials and methods

### Growth medium

Cells were grown in either CTFUD rich medium [15] (Additional file 1: Table S3) or MTC-5 chemically defined medium [48] (Additional file 1: Table S4).

### Strain construction

Targeted genetic modifications of *C. thermocellum* were performed as previously described [15]. Strain LL1590 was constructed by deleting the native *C. thermocellum adhE* gene in LL1592. Strain LL1679 was constructed by introducing the *Sulfolobus solfataricus gapN* gene at the *ldh* locus in strain LL1592. Strain LL1707 was constructed by introducing the *Thermoanaerobacterium saccharolyticum gapDH* gene into the *ldh* locus in LL1592. Complete genomes of constructed strains can be reconstructed from resequencing data presented in Table 2. This data is also accessible from the NCBI Sequence Read Archive using the BioProject accession number PRJNA986549.

**Table 2:** Strains used in this work

Strain ID	Description	Sequence data accession	Reference
M1442	*Thermoanaerobacterium saccharolyticum* engineered for production of ethanol at high yield and titer. Also known as strain LL1049	SRP052455	[8]
LL1004	WT *C. thermocellum* DSM 1313 from DSMZ culture collection	NCBI reference sequence NC_017304.1	
LL1570	*C. thermocellum* expressing a heterologous ethanol production pathway from *T. saccharolyticum* ∆hpt ΔreIII P2638::adhA(Tsc) nfnAB(Tsc) adhEG544D(Tsc) Clo1313_2637::Ptsc0046-pforA(Tsc)-ferredoxin(Tsc) ∆pfor1 ∆pfor4 ∆pfor3 ∆pfor2 ∆pfor5	SRX4014213	[3]
LL1590	LL1592 ∆*adhE*	SRX5290158	(This work)
LL1592	LL1570 ∆*ldh*	SRX5290154	(This work)
LL1679	LL1592 ∆*ldh*::*Ss_gapN*	SRX20750948	(This work)
LL1707	LL1592 ∆*ldh*::*Tsc_gapDH*	SRX9406543	(This work)
LL1731	LL1679 after adaptation in the presence of added ethanol (population)	SRX9409525	(This work)
LL1732	LL1707 after adaptation in the presence of added ethanol (population)	SRX9409524	(This work)
LL1733	LL1592 after adaptation in the presence of added ethanol (population)	SRX9409526	(This work)
LL1787	Single colony isolate from LL1731 population	SRX20750950	(This work)
LL1806	Single colony isolate from LL1732 population	SRX20750956	(This work)
LL1789	Single colony isolate from LL1733 population	SRX20750951	(This work)
LL1727	LL1592 after adaptation in the presence of added ethanol (population)	SRX9642141	(This work)
LL1728	LL1592 after adaptation in the presence of added ethanol (population)	SRX9642195	(This work)
LL1729	LL1592 after adaptation in the presence of added ethanol (population)	SRX9642247	(This work)
LL1730	LL1592 after adaptation in the presence of added ethanol (population)	SRX9642241	(This work)
LL1734	LL1592 after adaptation in the presence of added ethanol (population)	SRX20750957	(This work)
LL1735	LL1592 after adaptation in the presence of added ethanol (population)	SRX9642242	(This work)
LL1736	LL1592 after adaptation in the presence of added ethanol (population)	SRX20750958	(This work)
LL1737	LL1592 after adaptation in the presence of added ethanol (population)	SRX20750959	(This work)
LL1761	LL1734 population after additional serial transfers (population)	SRX20750961	(This work)
LL1759	LL1735 population after additional serial transfers (population)	SRX20750964	(This work)
LL1760	LL1736 population after additional serial transfers (population)	SRX20750962	(This work)
LL1758	LL1737 population after additional serial transfers (population)	SRX20750960	(This work)
LL1792	Single colony isolate from population LL1761	SRX20750954	(This work)
LL1790	Single colony isolate from population LL1759	SRX20750952	(This work)
LL1791	Single colony isolate from population LL1760	SRX20750953	(This work)
LL1805	Single colony isolate from population LL1758	SRX20750955	(This work)

### Whole genome resequencing (WGS) at Dartmouth

Genomic DNA was prepared using the Omega E.Z.N.A. kit following the manufacturer’s protocol (Omega Bio-Tek, GA, USA). 500 ng of DNA was used for WGS library preparation using the NEBNext Ultra II FS DNA Library Prep Kit for Illumina (New England Biolabs, MA, USA). Fractionated, adapter ligated DNA fragments went through 5 rounds of PCR amplification and purification. The resulting WGS library was sequenced at the Genomics and Molecular Biology Shared Resource (GMBSR) at Dartmouth. Libraries were diluted to 4 nM, pooled and loaded at 1.8 pM onto a NextSeq500 Mid Output flow cell, targeting 130 million 2 × 150 bp reads/sample. Base-calling was performed on-instrument using RTA2 and bcls converted to fastq files using bcl2fastq2 v2.20.0.422.

### Whole genome resequencing (WGS) at JGI

Genomic DNA was submitted to the Joint Genome Institute (JGI) for sequencing with an Illumina MiSeq instrument. Paired-end reads were generated, with an average read length of 150 bp and paired distance of 500 bp. Unamplified libraries were generated using a modified version of Illumina’s standard protocol. 100 ng of DNA was sheared to 500 bp using a focused ultrasonicator (Covaris). The sheared DNA fragments were size selected using SPRI beads (Beckman Coulter). The selected fragments were then end repaired, A-tailed and ligated to Illumina compatible adapters (IDT, Inc) using KAPA Illumina library creation kit (KAPA biosystems). Libraries were quantified using KAPA Biosystem’s next-generation sequencing library qPCR kit and run on a Roche LightCycler 480 real-time PCR instrument. The quantified libraries were then multiplexed into pools for sequencing. The pools were loaded and sequenced on the Illumina MiSeq sequencing platform utilizing a MiSeq Reagent Kit v2 (300 cycle) following a 2 × 150 indexed run recipe.

### WGS data analysis

Read data was analyzed with the CLC Genomic Workbench version 22 (Qiagen Inc., Hilden, Germany). First, reads were trimmed using a quality limit of 0.05 and ambiguity limit of 2. Then 2.5 M reads were randomly selected (to avoid errors due to differences in the total number of reads). Reads were mapped to the reference genome (NC_017304). Mapping was improved by two rounds of local realignment. The CLC Basic Variant Detection algorithm was used to determine small mutations (single and multiple nucleotide polymorphisms, short insertions and short deletions). Variants occurring in less than 35% of the reads or fewer than 4 reads were filtered out. The fraction of the reads containing the mutation is presented in Additional file 1: Table S1. To determine larger mutations, the CLC InDel and Structural Variant algorithm was run. This tool analyzes unaligned ends of reads and annotates regions where a structural variation may have occurred, which are called breakpoints. Since the read length averaged 150 bp and the minimum mapping fraction was 0.5, a breakpoint can have up to 75 bp of sequence data. The resulting break- points were filtered to eliminate those with fewer than ten reads or less than 20% “not perfectly matched.” The breakpoint sequence was searched with the Basic Local Alignment Search Tool (BLAST) algorithm [49] for similarity to known sequences. Pairs of matching left and right breakpoints were considered evidence for structural variations such as transposon insertions and gene deletions. The fraction of the reads supporting the mutation (left and right breakpoints averaged) is presented in Additional file 1: Table S1. Mutation data from CLC was further processed using custom Python scripts (https://github.com/danolson1/cth-mutation).

### Adaptation strategies

Cells were grown at 55 C in a COY anaerobic chamber (Coy Laboratory Products, Grass Lake, MI). Serial transfers were performed in either rich medium (lineage group A) or chemically defined medium (lineage groups B and C) in medium with 50 g/L cellobiose, using different transfer strategies described in Table 1. Transfers were approximately 1% by volume (100 μl into 10 ml), which allows for approximately 6.6 generations per transfer. The effective population size was approximately 1e8 (0.1 ml of an OD_600_ = 1 culture with 1e9 cells/ml/OD).

### Ethanol tolerance assay

Ethanol tolerance was determined by measuring the maximum specific growth rate (μ_max_). 2 µl of frozen cells was inoculated 198 µl of media containing various concentrations of ethanol. Assays were performed in a 96-well plate with a ThermalSeal RTS Sealing Film (Sigma part number Z742256). We tested several sealing films to minimize ethanol evaporation, and these sealing films performed significantly better than others we tested. Ethanol concentration in each well was measured both before and after the growth assay using an enzyme-linked assay we have recently developed (10.17504/protocols.io.brvcm62w) (10.17504/protocols.io.brvcm62w). Wells showing significant ethanol evaporation were excluded from analysis. Cell growth was determined by measuring the absorbance at 600 nm. Absorbance was measured at 6 min intervals for 96 h. The specific growth rate was determined by measuring the maximum slope of the log-transformed and blank-subtracted absorbance data. The slope was determined using a sliding window over 40 points (4 h). For cells adapted in defined medium (MTC-5, lineage groups B and C), ethanol tolerance was measured in MTC-5 medium with 5 g/L cellobiose. For cells adapted in rich medium (CTFUD, lineage group A), ethanol tolerance was measured in CTFUD medium with 5 g/L cellobiose.

### Bottle fermentations

High substrate (50 g/L cellobiose) fermentations were performed in 150 ml sealed serum bottles with 20 ml working volume. The working volume was chosen to limit the maximum pressure for safety reasons. For fermentations with added ethanol, anhydrous ethanol was used (Sigma 459836).

### Protein purification

*E. coli* strains were routinely cultured from frozen glycerol stocks in solid LB medium (Fisher) supplemented with appropriate antibiotics. Liquid cultures were grown aerobically in TB medium (Sigma-Aldrich) with the appropriate antibiotic to mid-exponential phase (optical density at 600 nm =  ~ 0.5) with shaking at 225 rpm at 37 °C.

Once the cultures reached the mid-exponential phase, 0.2 mM IPTG (Sigma) was added to the culture to induce protein expression and incubated at 16 °C with shaking at 225 rpm for 18 h. Afterwards, induced cultures were transferred to serum bottles and purged with N_2_ to generate an anaerobic protein expression environment. Cultures were incubated for a further 3 h with shaking at 225 rpm at 30 °C before harvest.

All the subsequent steps were carried out anaerobically in an anaerobic glove box (Coy). Cells were harvested by centrifugation at 7000*g* for 15 min. The spent culture was discarded and pellet cells were washed once with Tris Buffer (50 mM, pH 8.34). Pellet cells were stored anaerobically at − 80 °C.

Prior to protein purification, the frozen pellets were thawed on ice and resuspended in 1 ml B-PER (Thermo Scientific) with Ready-Lyse Lysozyme and OmniCleave Endonuclease (Biosearch Technologies). Cell lysate was centrifuged at 13,000*g* for 5 min at room temperature to remove cell debris. The supernatant containing His-tagged protein was applied directly to a Ni–NTA–agarose purification column (His SpinTrap; Cytiva) then subjected to anaerobic affinity column purification according to the manufacturer’s protocol. Briefly, the column was first equilibrated with binding buffer (60 mM imidazole) and then cell lysate was applied to the column. Next, the column was washed twice with binding buffer (60 mM imidazole) and thrice with wash buffer (80 mM imidazole). The His-tagged protein was eluted with elution buffer (200 mM imidazole). Purified His-tagged enzymes were stored on ice. An *E. coli* strain harboring the pCB17 plasmid, which expresses the WT *C. thermocellum* AdhE protein, was used as a control to measure ADH or ALDH activity.

### Protein quantification

Protein concentration was measured using the Bradford assay (Fischer Scientific Catalog No. PI23238), based on the change in color of Coomassie G-250 measured at 595 nm. Bovine Serum Albumin (BSA) was used as the standard.

### Cell-free extract (CFE) preparation

To prepare cell-free extracts for enzyme assays, cells were grown to mid-log phase in either chemically defined medium (lineage groups B and C) or rich medium (lineage group A). Cells were harvested by centrifugation, the supernatant was decanted, and the cell pellets were stored at −80 °C. On the day of the assay, cell pellets were thawed at room temperature and resuspended in a small volume (100–200 μl) of enzyme assay buffer (described below). Concentrated ReadyLyse enzyme (Lucigen R1804M, ~ 1 μl) was added to the resuspended cells and they were incubated at room temperature until an increase in viscosity was observed, usually 10–20 min. Then 1–2 μl DNAseI (New England Biolabs M0303S) was added to reduce the viscosity. After an additional 5–10 min incubation at room temperature, the suspension was centrifuged for 3 min at maximum speed (12,000 ×*g*) and the supernatant was collected as cell-free extract (CFE) for use in subsequent enzyme assays.

### Enzyme assays

To maintain anaerobic conditions, enzyme assay stocks were weighed aerobically, then brought into the anaerobic chamber and resuspended with water that had been autoclaved to remove oxygen. Concentrations of NADH and NADPH were verified by spectrophotometer immediately prior to the assay. All enzyme assays were performed in enzyme assay buffer (100 mM Tris–HCl, 250 mM NaCl, 2 mM MgCl_2_, 1 mM dithiothreitol, 10 mM sodium ascorbate, and 0.5 mM ammonium ferrous sulfate. The final pH was 7.5). The addition of sodium ascorbate and ammonium ferrous sulfate was intended to prevent inactivation of the ADH domain [50].

Enzyme assays were performed in a 60 μl reaction volume in a 384 well plate. For each sample, several 2-fold  dilutions were prepared in enzyme assay buffer with the addition of 0.03 mg/ml BSA protein. Enzyme assays were performed at 40 °C. This temperature is lower than the usual growth temperature of 55 °C, but was chosen due to experimental constraints related to performing enzyme assays in a multi-well plate format. At 55 °C, the spontaneous rate of NADH and NADPH degradation increases, which requires assays to be run with higher concentrations of enzyme. At 55 °C, more time is needed for the temperature of the 384 well plate to equilibrate, which requires assays to be run with lower concentrations of enzyme. As a result of these competing constraints, it is not possible to accurately measure enzyme activity in a 384 well plate at 55 °C (note that this is more of a problem for NADPH-linked activity than NADH-linked activity). Furthermore, a temperature of 40 °C has been previously used for *C. thermocellum* enzyme assays [51]. Assay plates were sealed with a ThermalSeal RTS Sealing Film. The concentration of NADH or NADPH was determined by measuring the absorbance at 340 nm. NADH and NADPH standard curves were included in each assay plate. Absorbance was measured at 15 s intervals for 3.5 h.

The acetaldehyde dehydrogenase (ALDH, EC 1.2.1.10) enzyme assay included 0.45 mM NADH or NADPH and 1 mM acetyl-CoA (final concentration). The alcohol dehydrogenase (ADH, EC 1.1.1.1 or 1.1.1.2) enzyme assay included 0.45 mM NADH or NADPH and 10 mM acetaldehyde (final concentration).

### Supplementary Information

**Additional file 1:** **Table S1.** List of all of the mutations identified in the strains described in this work. **Table S2.** Fermentation data from strains grown in the presence or absence of 10 g/L added ethanol. **Table S3.** Recipe for CTFUD rich medium. **Table S4.** Recipe for MTC-5 chemically defined medium.

## Data Availability

Data availability is described the materials and methods. Data and materials not present in public repositories or in the supplemental data are available upon request to the corresponding author, Daniel G. Olson.
